# Effects of Water and Alkaline Solution on Durability of Carbon-Glass Hybrid Fiber Reinforced Polymer Bars

**DOI:** 10.3390/polym13213844

**Published:** 2021-11-07

**Authors:** Yixun Yu, Yunfeng Pan, Ronggui Zhou, Xinbo Miao

**Affiliations:** School of Civil Engineering and Architecture, Zhejiang Sci-Tech University, Hangzhou 310018, China; 2017331200107@mails.zstu.edu.cn (Y.Y.); 2017331200110@mails.zstu.edu.cn (R.Z.); 2018331200211@mails.zstu.edu.cn (X.M.)

**Keywords:** fiber reinforced polymer bars, carbon fiber, water diffusion, interlaminar shear strength

## Abstract

The glass fiber reacts with the hydroxyl owing to the concrete pore solution. A thin coat of carbon fiber wraps around the internal GFRP bars to improve the durability of internal GFRP bars in harsh environments. This paper investigates the effect of a thin carbon fiber coat on the durability of the carbon–glass hybrid fiber reinforced polymer bars (HFRP bars) in water, and compares the performance of FRP bars in alkaline solution. To this end, the water absorption behavior, interlaminar shear strength of both the GFRP bars and the HFRP bars was characterized in water and alkaline solution. The results indicate that the diffusivity coefficient of the carbon fiber coat is higher than that of internal GFRP in water. Compared to the GFRP bars in water, the HFRP bars have a higher diffusivity coefficient and saturation water absorption. It caused that the interlaminar shear strength of the HFRP bars aged in water at a temperature of 60 °C for 140 days decreases more markedly than that of the GFRP bars aged under similar conditions. Finally, it was proved that the thin carbon fiber coat does not slow the deterioration of the GFRP bars in water, while the carbon fiber coat significantly improves the retention of the interlaminar shear strength of the HFRP bars in the alkaline solution owing to the prevention of internal glass fiber reactivated by alkali ions.

## 1. Introduction

Glass fiber reinforced polymer (GFRP) bars replaced steel bars have become popular as a reinforcement for the concrete exposed to harsh environments [1]. Compared to conventional steel, GFRP has a higher strength-to-weight ratio and superior resistance to chlorine [2]. The pore solution of concrete is an alkaline solution produced by the leaching of alkalis owing to the hydration of cement. Although GFRP bars are immune to chlorine-induced corrosion, the glass fibers erode through the chemical reaction with alkalis [3]. Therefore, a thin coat of carbon fiber wraps around the internal GFRP bars, and the durability of the carbon–glass hybrid fiber-reinforced polymer (HFRP) bars in alkaline solution is largely improved [4]. In fact, the HFRP bars in concrete are exposed to water molecules in addition to the alkali ions. However, the effect of a thin carbon fiber coat on the diffusion of water into HFRP bars and on the mechanical properties of the HFRP bars aged in water has not well been investigated.

The diameter of the fibers influences the water diffusion in fiber reinforced polymers (FRPs) [5,6,7]. The fibers act as a barrier in composites and induce the tortuosity of the diffusion path. The larger the tortuosity of the diffusion path, the slower the water diffusion rate. The thickness of the fiber–matrix interface varies with the diameter of the fibers. It was reported that the diameter of glass fiber and carbon fiber is 28 μm and 7 μm, respectively [4]. The thickness of the interface between carbon fibers and an epoxy matrix is around 100 nm, while that of the interface between glass fibers and an epoxy matrix ranges from 100 to 300 nm [8]. Compared with glass fibers, carbon fibers with a smaller diameter provide a higher interface area per unit volume and thus more possible pathways for the rapid diffusion of water, which accelerates the diffusion process of water [9]. It is also reported that the diffusivity coefficient of CFRP is 50% larger than that of GFRP [9].

The GFRP bars deteriorate with an increase in water absorption, which results in the degradation of their mechanical properties [10,11]. Indeed, water molecules are absorbed by the matrix resin owing to the hydroxyl or other polar groups [12], which leads the matrix resin to plasticize and the mechanical properties of the matrix to degenerate [13,14]. The reduction in the interlaminar shear stress for GFRP bars increased 20% with the 120% increase of the water absorption in the simulated normal pore concrete solution [3].

The interface between the fibers and the epoxy matrix plays a significant role in the long-term durability of the mechanical performance of fiber reinforced polymer [15,16]. The bonding between the fibers and the epoxy matrix depends on the physical adsorption and the chemical interactions such as van der Waals interactions, hydrogen bonding, and mechanical interlocking [17]. In the case of the chemical interactions, compared to the van der Waals interactions, hydrogen bonding plays a more significant role in the bond strength of the interface. The results of molecular dynamics show that the saturation water absorption causes a 58% reduction in the surface free energy involved with the hydrogen bonding [18]. Moreover, compared with dry conditions, moisture reduces the adhesion at the interface between the glass fibers and the epoxy by 24% [19].

Compared to composite materials consisting of one type of fiber, HFRP bars have a weak interface between the outer coat of the CFRP and the inner core of GFRP [20,21]. It is reported that the degradation of the interlaminar shear strength of HFRP bars depends on the level of water absorption and increases as the exposure time and temperature rise [20]. For example, the interlaminar shear strength of HFRP bars exposed to water for 32 weeks declines by 13% and 20% at a temperature of 40 and 60 °C respectively [20].

Considering the ratio of the area of the thin carbon layer to the section area of the HFRP bars (~0.3), the fiber hybridization effect is ignored in the present study. This work investigates the effect of a thin carbon fiber coat on the interlaminar shear strength of the HFRP bars exposed to water. Moreover, it examines the interlaminar shear strength of the GFRP bars and the HFRP bars aged in water and in alkaline solution. The degradation mechanism of the GFRP bars and the HFRP bars in the alkaline solution and water is also discussed.

## 2. Materials and Methods

### 2.1. Raw Materials

**Figure 1:**
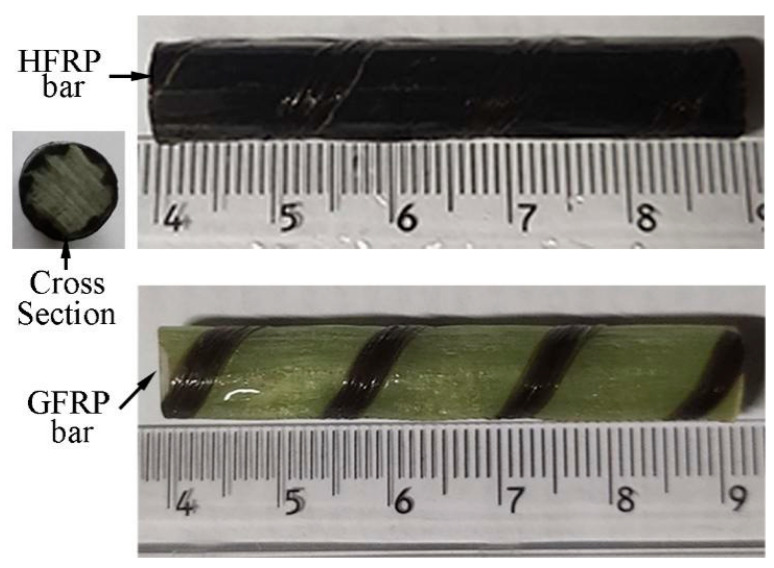
The image of GFRP and HFRP bars.

Figure 1 shows the GFRP bars and HFRP bars of the present study. The nominal diameter of GFRP bars and HFRP bars is 8mm. The characteristics of raw materials were provided by the manufacturer. The diameter of carbon fiber and E-glass fiber are 7 and 28 μm, respectively. The tensile strength of the carbon fiber and E-glass fiber are 4900 and 2200 MPa, respectively. The resin matrix is composed of Bisphenol-A resin and Methylhexahydrophthalic Anhydride hardener. The detailed fabrication process of HFRP bars was depicted by Pan and Yan [4]. Figure 1 shows the radius of the internal GFRP is varied along the interface between internal GFRP and carbon fiber coat. The cross-section of the HFRP bars was imaged, and imported into AutoCAD software. The areas of the internal GFRP and carbon fiber coat were calculated, respectively, and the ratio of the area of carbon fiber coat to internal GFRP was 0.3. The mean radius of the internal GFRP and thickness of the carbon fiber coat were determined to be 3.37 mm and 0.73mm.

The dynamic mechanical analysis (DMA) was adopted to measure the glass transition temperature (*T*_g_) of FRP bars. The three-point bending mode was applied, and the *T*_g_ of GFRP bars and HFRP bars were determined to be 165 °C and 159 °C, respectively. The glass transition temperature of the resin is determined to be 135.5 °C.

### 2.2. Exposure Conditions

**Figure 2:**
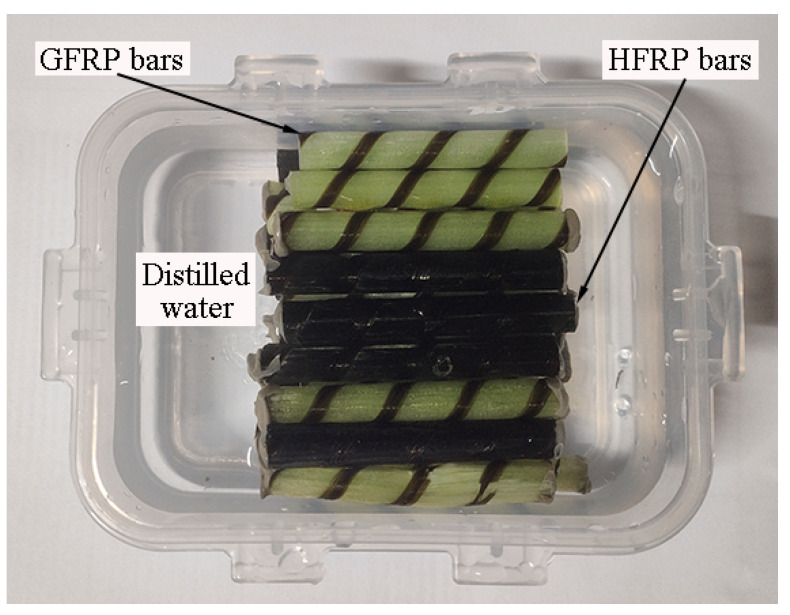
FRP bars immersed in the distilled water.

Figure 2 depicts the FRP bars immersed in distilled water. The ends of the FRP bars were sealed by epoxy adhesive to prevent water diffusion along the fiber direction. All of the specimens were dried at 60 °C in the oven for one day before immersion in water. After being immersed in the distilled water for specified periods, the short beam shear and their water absorption test were studied. It was reported that the environmental temperature below *T*_g_ accelerates the water absorption of FRPs, and does not change the mechanism of water absorption. Water baths at room temperature (~21 °C), 40 °C, and 60 °C were chosen in the present study. The chosen environmental temperature is less than that of the glass transition temperature of the resin.

The FRP specimens were labeled as follows. GTD0 and HTD0, respectively, represent the control samples of the GFRP bars and the HFRP bars without being immersed in distilled water. In the case of HT21 and GT21, the letters H and D represent the HFRP bars and GFRP bars, respectively, and “21” means the specimens immersed at 21 °C.

### 2.3. Moisture Uptake

The water uptakes of the GFRP bars and the HFRP bars were examined at a temperature of 21 °C, 40 °C, and 60 °C. For each temperature, weight gains of 10 replications with a length of 50 mm were weighed at regular intervals. The water absorption of the GFRP bars and the HFRP bars was calculated by [22]:

(1) $$M_{i}=\frac{W_{i}-W_{0}}{W_{0}}\times 100$$

where *M_i_* is the water absorption (%). $W_{i}$ denotes the mass (g) of aged specimen mass with the tested number *i*. $W_{0}$ indicates the control specimen mass (g).

The equation of Fick’s law can describe the water diffusion in FRPs [20]. The equation of Fick’s law is

(2) $$\frac{M_{t}}{M_{\infty }}=1-\sum_{n=1}^{\infty }\frac{4}{r^{2}\alpha_{n}^{2}}\exp(-D_{r}\alpha_{n}^{2}t)$$

where $M_{t}$ is the water absorption of the FRP at time *t*. $M_{\infty }$ indicates the saturation water absorption, *r* denotes the radius of the FRP, $\alpha_{n}^{}$ represents the *n*th root of the Bessel function of zero order, and $D_{r}$ is the radial diffusion coefficient perpendicular to the fiber direction (mm^2^/s).

### 2.4. Finite Element Method for Diffusion of Moisture into FRP Bars

**Figure 3:**
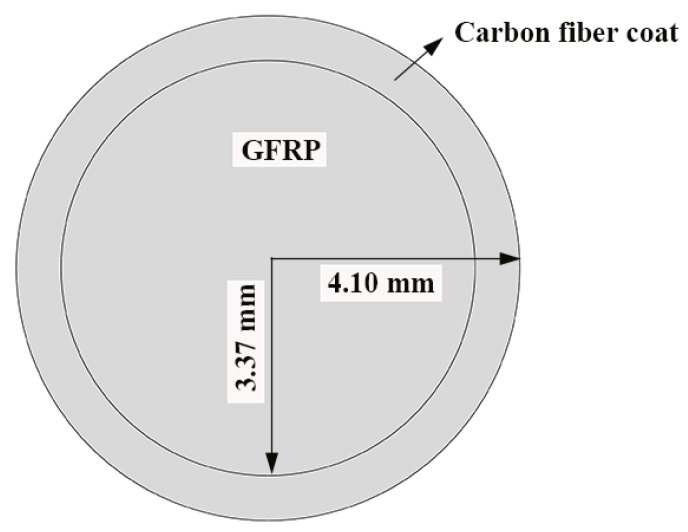
The finite element model of the diffusion of water into the HFRP bars.

The water absorption behavior of the HFRP bars depends on the water diffusion of the internal GFRP and the carbon fiber coat. Since the water diffusion behavior of the carbon fiber coat differs from that of the internal GFRP, the diffusivity coefficient of the carbon fiber coat cannot be determined based on the present experimental results. Thus, the inverse analysis was employed to determine the diffusivity coefficient of the carbon fiber coat by using Abaqus software. To this end, the modulus of mass diffusion was adopted, and the length of the unidirectional FRP bars was several times larger than their diameter. In the present study, the water diffusion perpendicular to the fiber direction was assumed. The values of the parameters of diffusivity and solubility were set to be the diffusivity coefficient and the saturation water absorption of the materials, respectively. The diffusivity coefficient and the saturation water absorption for HFRP and the GFRP bars were determined by a water absorption test. Figure 3 illustrates the 2-D finite element model of the transverse section of the HFRP bars; the quadratic element (DC2D6) was used for the composite geometry [5]. The water absorption of the FRP bars was determined by the arithmetic average of the element centroid. The boundary conditions of the HFRP bars were defined as 100% moisture concentration on the outer surface of the carbon fiber coat.

### 2.5. Short Beam Shear Test

**Figure 4:**
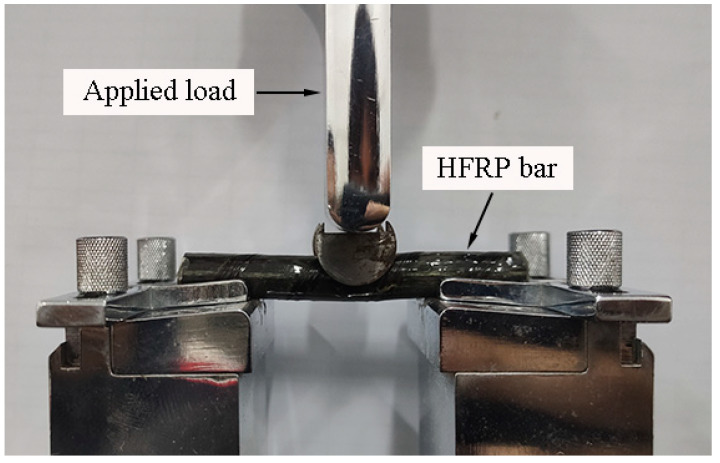
The image of short beam shear test setup.

The short beam shear test was adopted to characterize the evolution of interlaminar shear strength [23]. The HFRP bars were tested by using the short beam shear test in Figure 4. As recommended by the authors of [4], the span-to-diameter ratio was set at four in this work. The load was applied at a displacement rate of 1.3 mm/min by using the electronic universal testing machine [23].

The following equation was adopted to calculate the interlaminar shear strength.

(3) $$\tau_{\max}=0.849P/D^{2}$$

where $\tau_{\max}$ is the maximum shear stress of FRP bars (MPa). *P* represents the peak load (kN). *D* indicates the nominal diameter of the FRP bars (mm).

## 3. Results and Discussion

### 3.1. Weight Change

**Figure 5:**
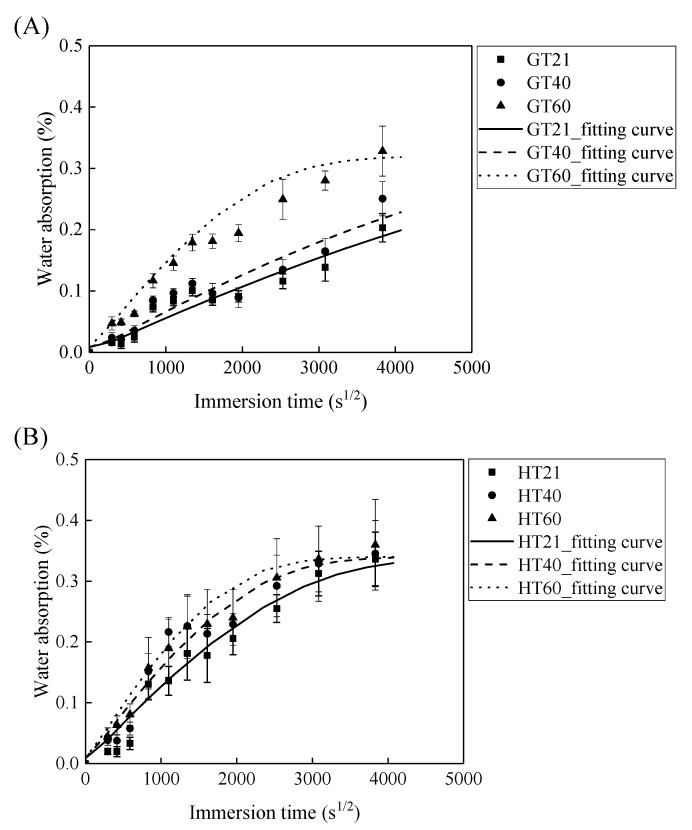
The saturation water absorption of (**A**) the GFRP bars and (**B**) the HFRP bars.

Figure 5 demonstrates that the water absorption of the GFRP bars and the HFRP bars first increases with the immersion time and then levels off. The parameters of $D_{r}$ and $M_{\infty }$ are fitted by Equation (2) and are listed in Table 1. According to this table, the radial diffusion coefficient enlarges with the exposure temperature. For instance, the radial diffusion coefficient of the GFRP bars at a temperature of 40 and 60 °C is respectively 35% and 500% larger than that at a temperature of 21 °C. Moreover, the bound water strongly depends on the exposure temperature, and a higher exposure temperature results in a higher rate of diffusion of water into the epoxy matrix [24]. The radial diffusion coefficient of the GFRP bars at a temperature of 60 °C is 3.45 times larger than that at a temperature of 40 °C.

In addition, at a similar exposure temperature, the radial diffusion coefficient of the HFRP bars is larger compared to GFRP bars. For example, the radial diffusion coefficient of the HFRP bars is 2.6 and 3.4 times larger than that of the GFRP bars at a temperature of 21 and 40 °C, respectively. Therefore, the presence of the carbon fiber coat increases the diffusion of water into the HFRP bars. Moreover, an elevated temperature of 60 °C further deteriorates the bond between the carbon fiber coat and the internal GFRP; thus the diffusion coefficient of the HFRP bars is 11% larger compared to GFRP bars at a temperature of 60 °C.

Owing to the insufficient exposure period in this study, the saturation water absorption of the FRP bars at a temperature of 21 and 40 °C does not reach a plateau. The saturation water absorption of an epoxy matrix is dependent on its chemical structure rather than the exposure temperature [25]. The saturation water absorption is, therefore, assumed to be independent of the exposure temperature in the current work. The saturation water absorption calculated by Equation (2) is listed in Table 1. The calculated saturation water absorption of the GFRP bars and the HFRP bars is 0.29% and 0.33%, respectively. The water absorption test of the epoxy used in the present study shows that a 0.97% saturation water absorption reduced the *T*_g_ of neat epoxy from 135.5 °C to 132.4 °C [26]. The water absorption of FRP bars is mainly caused by the water absorption of the epoxy matrix. It was reported that the saturation water absorption of neat epoxy is 0.97% [26], and the predicted saturation absorption of the GFRP bars with the fiber volume fraction of 60% is determined to be 0.23% by the water absorption of the epoxy matrix [5]. The predicted water absorption of GFRP bars is similar to the tested result (0.29%). The saturation water absorption of the HFRP bars is larger compared to GFRP bars. The larger of the saturation water absorption of HFRP was attributed to the higher void content of HFRP bars. The higher void content of HFRP bar was caused by the weak bond between internal GFRP and carbon fiber coat.

### 3.2. Determination of Radial Diffusivity Coefficient of Carbon Fiber Coat

The diffusivity coefficient and saturation water absorption of the FRP bars can be determined by Equation (2), while it is difficult to obtain the saturation water absorption and diffusivity coefficient of the carbon fiber coat. To determine the diffusivity coefficient and saturation water absorption of the carbon fiber coat, a numerical analysis was conducted using Abaqus software. It was assumed that the water molecules are chiefly absorbed by the epoxy matrix in the FRP bars. Thus, the water absorption of the HFRP bars can be expressed in:

(4) $$\frac{m_{\mathrm{wg}}+m_{\mathrm{wc}}}{M_{c}+M_{g}}=M_{H\infty }$$

where $m_{\mathrm{wg}}$ and $m_{\mathrm{wc}}$ are the water mass gain of the inner GFRP and the outer carbon fiber coat respectively (g); *M*_c_ and *M*_g_ indicate the mass of the inner GFRP and the outer carbon fiber coat respectively (g). $M_{H\infty }$ is assumed to be the saturation water absorption of the HFRP bars and equals 0.33% (see Table 1).

(5) $$M_{i}=\rho_{i}V_{i}$$

where ρ (g/mm^3^) and *V* (mm^3^) represent the density and volume of the specimens respectively. *i* indicates either the carbon fiber coat (*c*) or the GFRP (*g*). The density of the E-glass fibers and the carbon fibers are set to be 2.54 and 1.8 g/cm^3^, respectively.

By substituting Equation (5) into Equation (4), the saturation water absorption of the carbon fiber coat can be defined as:

(6) $$\frac{m_{\mathrm{wc}}}{M_{c}}=0.463$$

**Figure 6:**
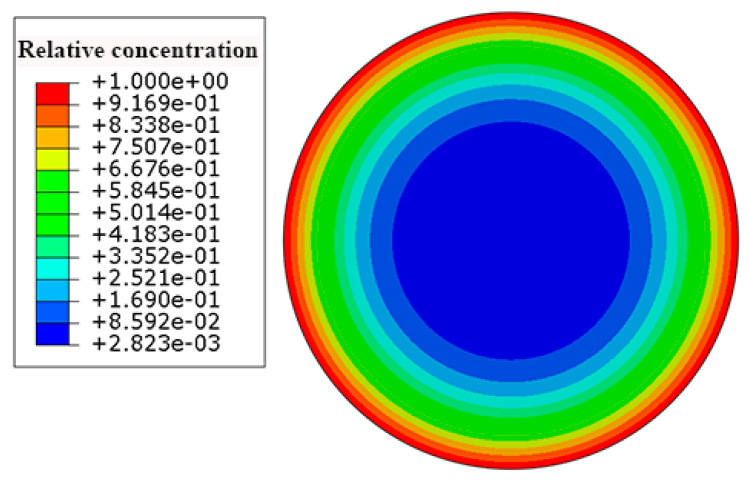
The distribution of the concentration of the water in the HFRP bars.

According to Equation (6), the saturation water absorption of the carbon fiber coat was set at 0.463. Figure 6 depicts the contours of the concentration of the water in the HFRP bars. The concentration of the water decreases from the surface to the center of the HFRP bars. As can be seen, the concentration of the water on the edge of the HFRP bars is higher than that close to the center of the HFRP bars.

**Figure 7:**
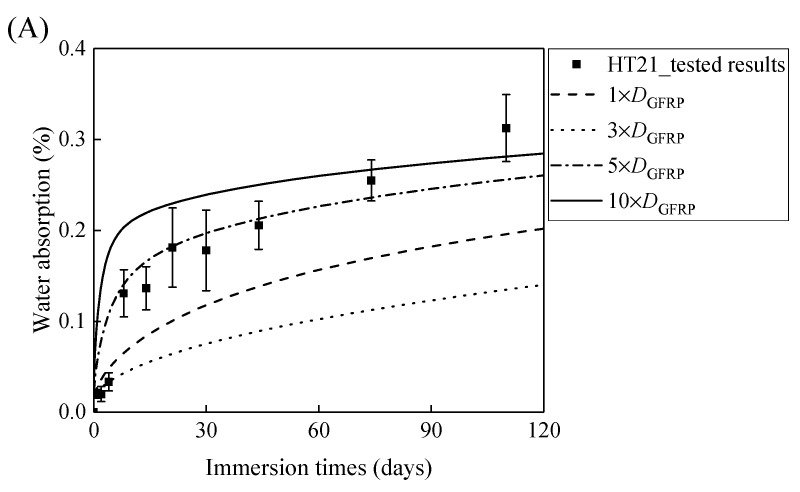

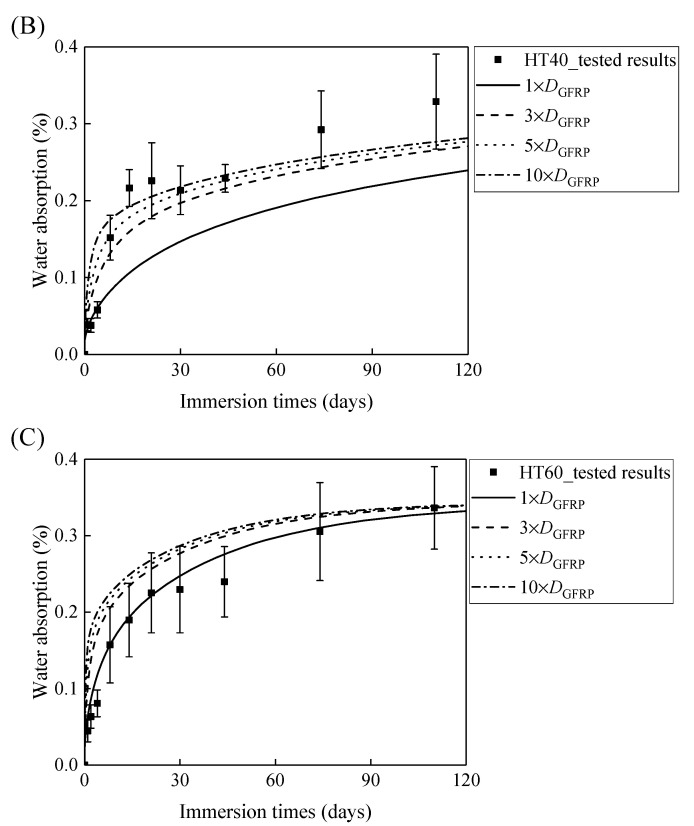
The variation in the water absorption of the HFRP bars with the immersion time at various temperatures for the determination of the radial diffusivity coefficient of the carbon fiber coat: (**A**) 21 °C, (**B**) 40 °C, and (**C**) 60 °C.

Figure 7A,B demonstrates that the curves of the water absorption of the HFRP bars with respect to the immersion period vary owing to the different radial diffusivity coefficient of the carbon fiber coat. For the specimens at a temperature of 21 °C, when the radial diffusivity coefficient of the carbon fiber coat is five times that of the internal GFRP bar, the simulated results match the experimental data. Moreover, at a temperature of 40 °C, the radial diffusivity coefficient of the carbon fiber coat is determined to be 10 times that of the internal GFRP bar. Nevertheless, at a temperature of 60 °C, the radial diffusivity coefficient of the carbon fiber coat equals that of the GFRP bar, which is attributed to the degradation of the interface bond between the outer carbon fiber coat and the inner GFRP bar, followed by the increase of the diffusivity coefficient of the internal GFRP. The radial diffusivity coefficient of the carbon fiber coat calculated at various temperatures is listed in Table 2.

### 3.3. Diffusion of Water Molecules into FRP Bars

Based on the diffusion parameters of the GFRP and the carbon fiber coat, the distribution of the water concentration can be expressed by [27]:

(7) $$\frac{\partial C(r,t)}{\partial t}=\frac{1}{r}\frac{\partial }{\partial t}\left[rD_{r}\frac{\partial C(r,t)}{\partial r}\right]$$

where C(r,t) is the radial distribution of the concentration of the water, and *t* indicates the water absorption time.

For the hybrid FRP, the boundary conditions are defined as:

(8) $$C=C_{0},\;r=b,\;t\geq 0$$

(9) $$C=C_{1},\;a<r<b,\;t=0$$

The solution to Equation (7), as a partial differential equation, can be determined through the separation of the variables and is given by:

(10) $$C_{c}(r,t)=1-\frac{2}{a}\sum_{n=1}^{\infty }\frac{J_{0}(\alpha_{n}r)}{J_{1}(a\alpha_{n})}\frac{1}{\alpha_{n}}\exp(-D_{\mathrm{rc}}\alpha_{n}^{2}t),\;0\leq r\leq a$$

(11) $$C_{g}(r,t)=1-\frac{2}{a}\sum_{n=1}^{\infty }\frac{J_{0}(\alpha_{n}r)(bJ_{1}(b\alpha_{n})-aJ_{1}(b\alpha_{n})}{b^{2}J_{1}^{2}(b\alpha_{n})-a^{2}J_{1}^{2}(a\alpha_{n})}\frac{1}{\alpha_{n}}\exp(-D_{\mathrm{rg}}\alpha_{n}^{2}t),\;a\leq r\leq b$$

where $D_{\mathrm{rc}}$ and $D_{\mathrm{rg}}$ are the radial diffusivity coefficient of the carbon fiber coat and the internal GFRP bar, respectively; *a* and *b* denote the radius of the internal GFRP bar and the HFRP bar, respectively; *J*_0_ and *J*_1_ represent the zeroth- and first-order Bessel function of the first kind, respectively.

**Figure 8:**
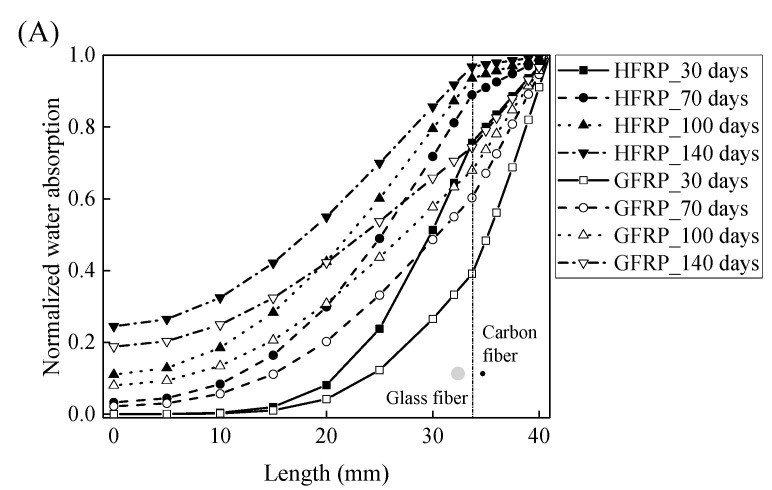

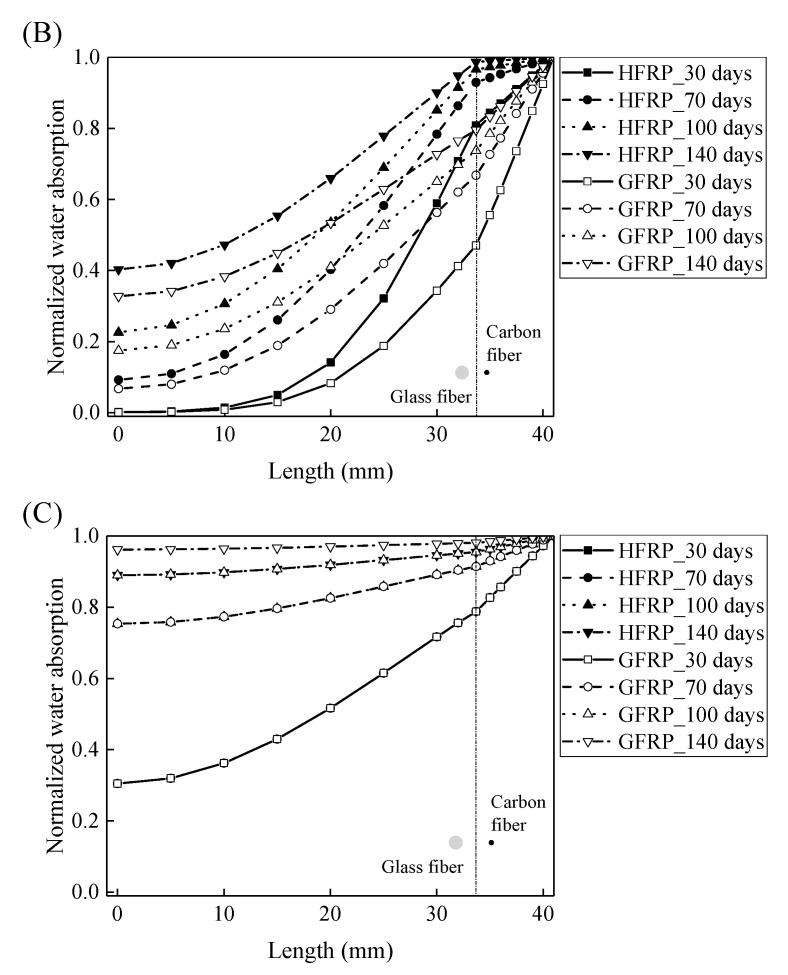
The variation in the concentration of the water with the length in the radial direction at a temperature of: (**A**) 21 °C, (**B**) 40 °C, and (**C**) 60 °C.

Using Equations (10) and (11), the concentration of the water is plotted with respect to the length at the various exposure temperatures in Figure 8. It is obvious that the concentration of the water declines with a decrease in the length. For the specimens at a temperature of 21 and 40 °C, the variation in the concentration of the water with the length can be distinguished between the GFRP bars and the HFRP bars. Furthermore, compared to GFRP bars, the water concentration of the HFRP bars is larger at each length. At a length of 3.1 mm, the water concentration of the HFRP bars is higher than that of the GFRP bars, which is attributed to the faster water absorption in the carbon fiber coat (3.1 mm < *r* < 4.1 mm). It is reported that the diffusion kinetics of fiber reinforced polymer depend on the relative concentration rather than the absolute concentration of moisture [5]. It results in more water in the internal GFRP for HFRP bars. Moreover, at a temperature of 60 °C, the variation in the concentration of water of the HFRP bars is similar to that of the GFRP bars. In fact, at a temperature of 60 °C, the coefficient of the diffusion of water into the internal GFRP bar increases and reaches the coefficient of the diffusion of water into the outer carbon fiber coat, which indicates that the carbon fiber coat wrapped on the GFRP bar cannot prevent the diffusion of water, and the water molecules diffuse into the FRP bars faster owing to the presence of the carbon fiber coat.

### 3.4. Interlaminar Shear Strength of FRP Bars

**Figure 9:**
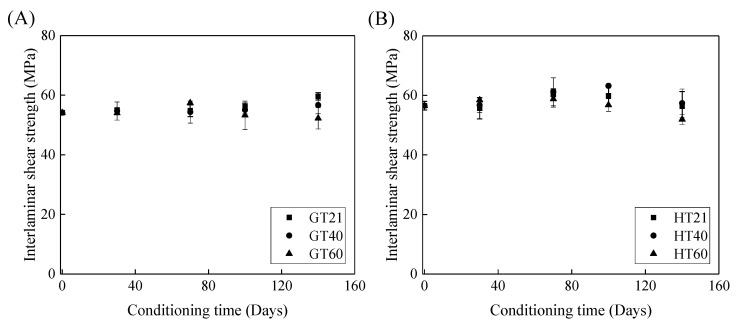
The effect of the immersion period on the interlaminar shear strength of (**A**) the GFRP bars and (**B**) the HFRP bars.

Figure 9 depicts the effects of the immersion time on the interlaminar shear strength of the FRP bars. Figure 9A shows that, compared to the control specimens, the interlaminar shear strength of the GFRP bars at a temperature of 21, 40, and 60 °C remains unchanged, increases by 5%, and declines by 3% respectively, which implies that both the post-curing and water-induced deterioration of the epoxy matrix occur within 140 days. With an increase in the exposure temperature, the impact of the deterioration of the GFRP bars on their interlaminar shear strength becomes more significant. Thus, the interlaminar shear strength of the GFRP bars is further reduced after 140 days of water immersion at 60 °C.

Figure 9B illustrates the effect of water immersion on the interlaminar shear strength of the HFRP bars. By increasing the immersion time to 100 days, the interlaminar shear strength of the HFRP bars slightly increases. Compared to the control specimens of the HFRP bars, the interlaminar shear strength of the HFRP bars after 100 days of immersion in water at 21, 40, and 60 °C increases by 5%, 11%, and 1%, respectively. Compared to the interlaminar shear strength of the specimens immersed in water for 100 days, extending the water immersion period by 40 days causes the interlaminar shear strength at 21, 40, and 60 °C to decrease by 6%, 9%, and 9%, respectively. Compared to the post-curing, the deterioration of the HFRP bars plays a more significant role in the interlaminar shear strength of the HFRP bars after 100 days of water immersion.

**Figure 10:**
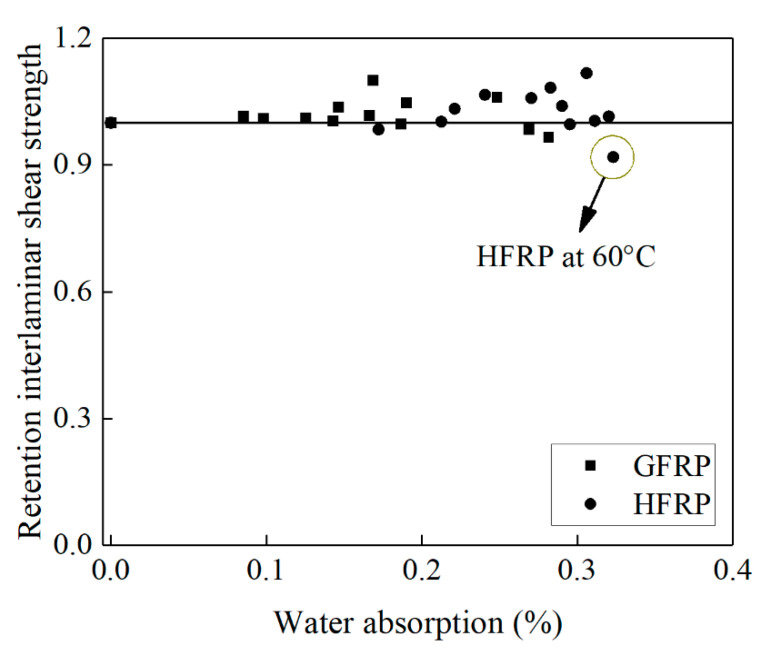
The effect of water absorption on the interlaminar shear strength of the FRP bars.

Figure 10 delineates the influence of water absorption on the interlaminar shear strength of the FRP bars. Compared to the control specimens, the interlaminar shear strength of the GFRP bars decreases by 2% until the water absorption reaches 0.27%, while the interlaminar shear strength of the HFRP bars declines by 8% until the water absorption reaches 0.32%. The presence of the carbon fiber coat results in the higher water absorption of the HFRP bars. The severest degradation of the HFRP bars at a temperature of 60 °C indicates the negative effect of the carbon fiber coat on the interlaminar bond strength of the GFRP bars in water (see Figure 10).

### 3.5. Comparison of Measured Results with Data in Literature

**Figure 11:**
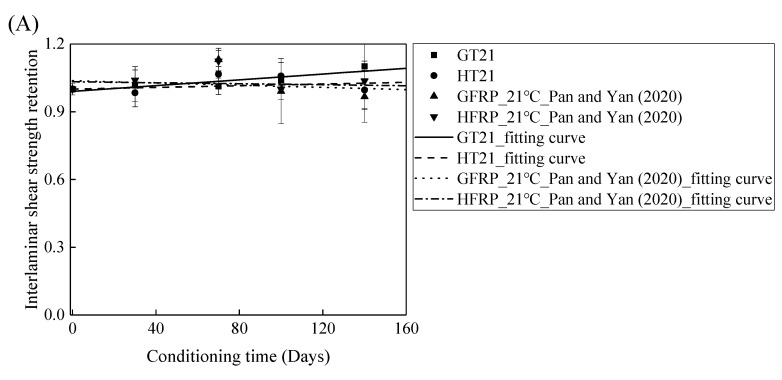

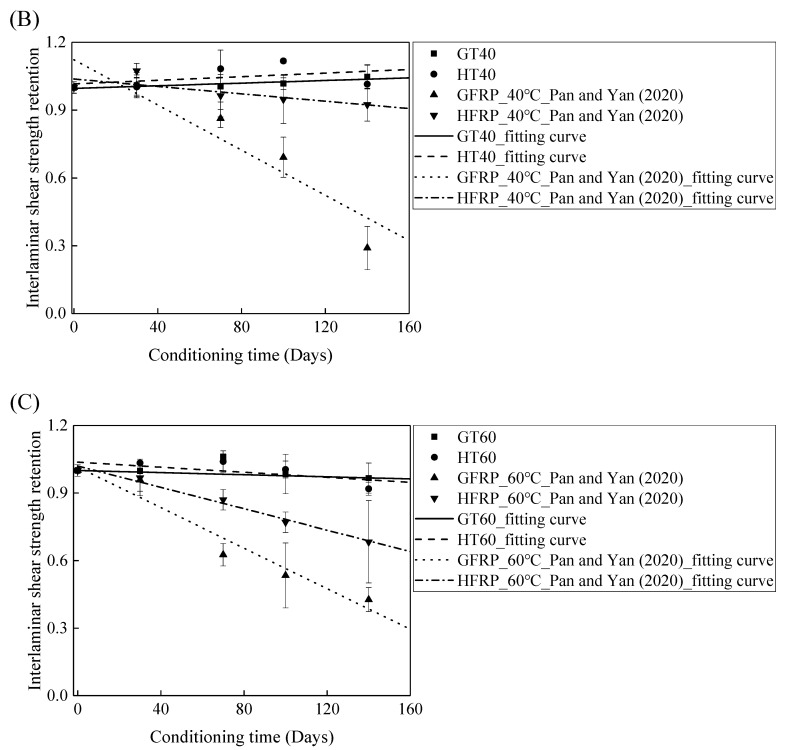
The comparison of the effects of the water and alkaline solution on the interlaminar shear strength of the FRP bars at a temperature of: (**A**) 21 °C, (**B**) 40 °C, and (**C**) 60 °C.

The FRP bars used in Ref. [4] and in the present study were from the same batch fabricated by Harbin FRP Institute of China. The effects of the alkaline solution with pH 13.4 on the interlaminar shear strength of the GFRP bars and the HFRP bars were investigated. Figure 11 compares the influence of the water and alkaline solution on the interlaminar shear strength of the FRP bars. Figure 11A demonstrates that the interlaminar shear strength of the GFRP bars in water for 140 days at a temperature of 21 °C increases by 10%, while the alkaline solution reduces the interlaminar shear strength of the GFRP bars by 3% under similar conditions. Moreover, the interlaminar shear strength of the HFRP bars immersed in water for 140 days at a temperature of 21 °C declines by 0.4%, while the interlaminar shear strength of the HFRP bars immersed in the alkaline solution increases by 4%. Therefore, the carbon fiber coat can slightly improve the resistance of the GFRP bars to the alkaline solution at a temperature of 21 °C.

Figure 11B depicts the variation in the interlaminar shear strength of the FRP bars in the water and alkaline solution at a temperature of 40 °C with the immersion period. For the specimens immersed in water, the interlaminar shear strength of the GFRP bars and the HFRP bars increases by 5% and 2%, respectively, which indicates that the post-curing of the epoxy matrix is dominated within 140 days of immersion. On the contrary, the alkaline solution reduces the interlaminar shear strength of the GFRP bars and the HFRP bars by 31% and 5%, respectively, after 100 days of immersion, which implies that the improvement effect of the carbon fiber coat applied on the GFRP bars is more significant in the alkaline solution than in water. Indeed, the carbon fiber coat inhibits the reaction of the glass fibers with the alkali ions and delays the degradation of the internal GFRP bar.

Figure 11C delineates the variation in the interlaminar shear strength of the FRP bars in water and alkaline solution at a temperature of 60 °C with the immersion period. It is obvious that the degradation of the interlaminar shear strength of the FRP bars is severer in the alkaline solution than in water. In fact, at an immersion period of 140 days, water reduces the interlaminar shear strength of the GFRP bars and the HFRP bars by 3% and 8%, respectively, while the alkaline solution lowers the interlaminar shear strength of the GFRP bars and the HFRP bars by 57% and 32% respectively, which indicates that the degradation of the FRP bars in water is due to the water absorption, and the deterioration process is slow. The carbon fiber coat enhances the rate of diffusion of water and results in a more serious deterioration of the HFRP bars in water. For the specimens immersed in the alkaline solution at a temperature of 60 °C, compared to GFRP bars, 58% larger for the retention of the interlaminar shear strength of the HFRP bars is found, which implies that the carbon fiber coat effectively prevents the chemical reaction of the alkali ions with the glass fibers and slows the deterioration of the internal GFRP bars.

### 3.6. Degradation Mechanism of FRP Bars

**Figure 12:**
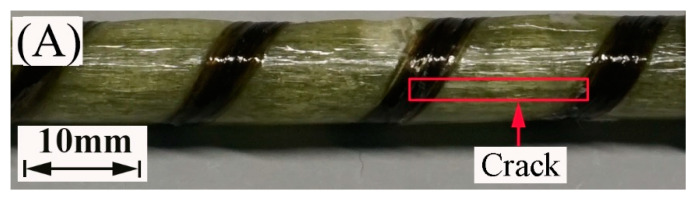

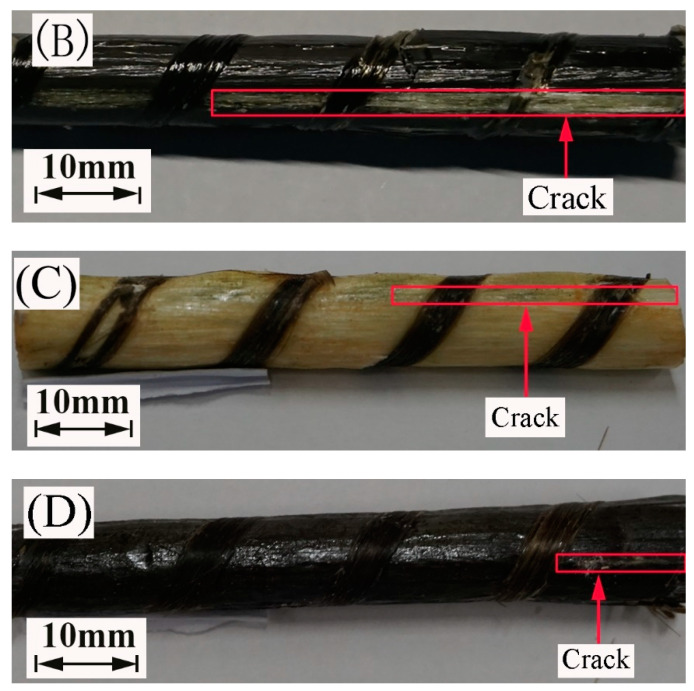
The failure mode of the specimens at a temperature of 60 °C: (**A**) the GFRP bars in water, (**B**) the HFRP bars in water, (**C**) the GFRP bars in the alkaline solution, and (**D**) the HFRP bars in the alkaline solution.

Figure 12 portrays the images of the failure mode of the FRP bars subjected to short beam shear testing. All the specimens crack along the neutral axis of the bar. Compared to the FRP bars immersed in water, the epoxy matrix on the surface of the FRP bars immersed in the alkaline solution hydrolyzes, leading to the appearance of the bare fibers. This is attributed to the reaction of the epoxy resin of the matrix with the alkali ions.

**Figure 13:**
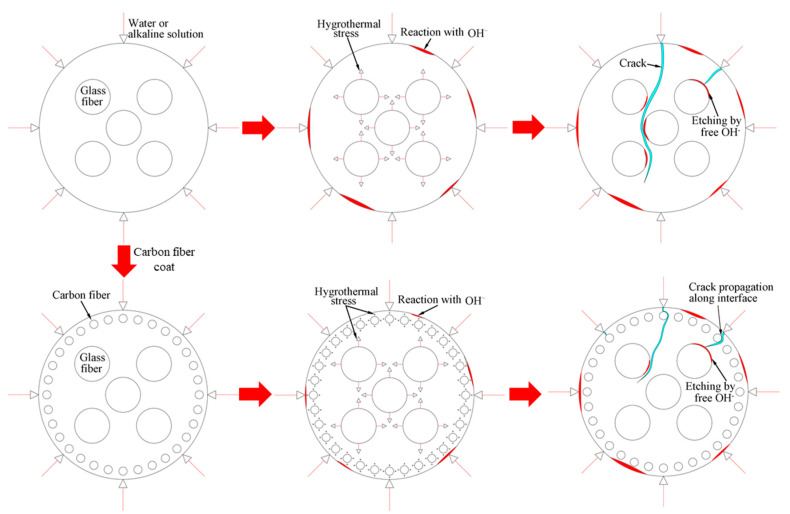
The degradation mechanism of the GFRP bars and the HFRP bars in water and the alkaline solution.

**Figure 14:**
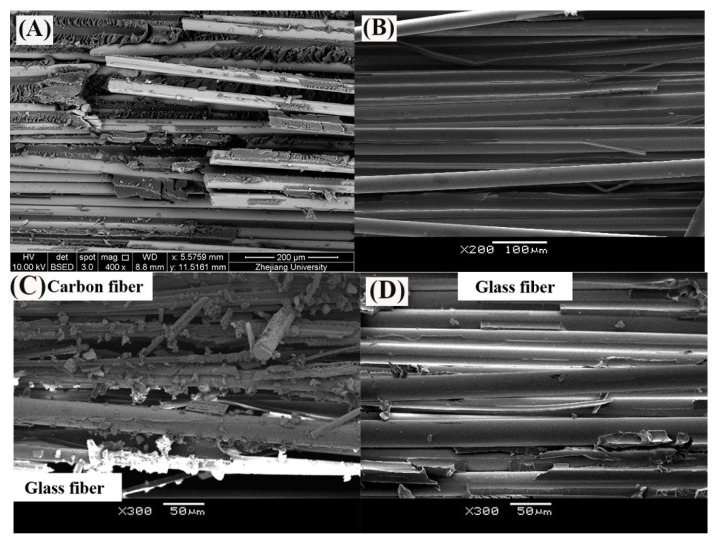
The SEM images of fracture surface of (**A**) GFRP bar at 60 °C in water [31], (**B**) GFRP bar at 60 °C in alkaline solution, (**C**) carbon fiber coat of HFRP bar at 60 °C in alkaline solution and (**D**) GFRP close to the CFRP/GFRP interface of HFRP bar at 60 °C in alkaline solution [4].

The deterioration mechanism of the FRP bars can reveal the correlation between the water absorption and the interlaminar shear strength of the FRP bars. Figure 13 illustrates the degradation mechanism of both the GFRP bars and the HFRP bars immersed in the water and alkaline solution. For the GFRP bars immersed in water, the epoxy matrix absorbs the water molecules and thus swells. On the one hand, this causes hygrothermal stress on the GFRP bars due to the mismatch between the coefficient of hygrothermal expansion of the epoxy matrix and that of the fibers. Once the hygrothermal stress exceeds the interfacial bond strength, interfacial cracking propagates with the immersion period and the exposure temperature due to the hygrothermal stress being larger than the interfacial bond strength. On the other hand, the swollen epoxy matrix is hydrolyzed. It is worth noting that the hydrolysis of the epoxy matrix is slower in water than in the alkaline solution. The failure mode of the coupled matrix failure and glass fiber fracture for the GFRP bars in water was found in Figure 14A, while interfacial debonding between glass fiber and epoxy matrix for the GFRP bars in alkaline solution is complete in Figure 14B. It means that the interfacial bond of GFRP bars in the alkaline solutions deteriorated more significantly than in water. The degradation of the epoxy resin results from the ester hydrolysis as shown below [28,29]:



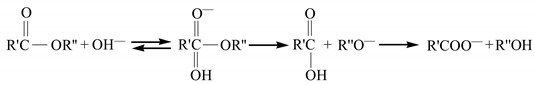

(12)


Once the alkali ions transfer along the cracks, the degradation of the E-glass fibers is largely caused by the leach of calcium with water molecules and the etching of the bulk glass with free OH^–^ [29]. The leaching and etching processes are as follows:



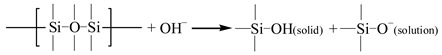

(13)




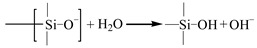

(14)


Compared to the original fiber structures, a gel-type product of the formation of the Si–OH is less dense and caused the reduction in the strength of the fiber–matrix interface. This gel also enhances the diffusion of water and alkalis, which further accelerates the degradation of the fibers [28].

To deal with the degradation of the glass fibers caused by their reaction with the alkali ions, a carbon fiber coat is wrapped on GFRP bars. It is proved that the carbon fiber coat improves the interlaminar shear strength of GFRP bars in alkaline solutions [4]. Firstly, the carbon fibers stop the propagation of cracks once a crack tip reaches a carbon fiber, and they lower the rate of crack propagation. Secondly, the HFRP bars deteriorate from the outer layer to the inner one. Although cracks propagate in the layer of the carbon fibers, the alkali ions cannot react with the carbon fibers. Figure 14C shows that the matrix failure occurred in the outer carbon fiber coat, and the interfacial debonding was found within GFRP close to the CFRP/GFRP interface in Figure 14D. Therefore, in the alkaline solution, the carbon fiber coat can slow the degradation of the HFRP bars, and cannot prevent the deterioration of the internal GFRP. Compared to GFRP bars, the retention of the interlaminar shear strength of the HFRP bars is larger. Nonetheless, the carbon fiber coat does not slow the diffusion of water into the HFRP bars. Conversely, the presence of the carbon fiber coat enhances the diffusion of water into the HFRP bars and increases their water absorption. This indicates that the larger the saturation water absorption of the FRP bars is, the severer the deterioration of the FRP bars becomes. It should be noted that the deterioration of the matrix, the fibers, and the interface between the matrix and the fibers are very slow in water compared to the alkaline solution. In fact, since the GFRP bars are embedded in concrete as a reinforcement for beams [30], they are chiefly exposed to alkaline conditions.

As discussed above, the durability of the HFRP bars is better than the GFRP bars in alkaline solution. The limitation of the study is that the FRP bars were directly immersed in a simulated solution. In fact, the FRP bars are embedded in concrete, and the alkalinity of the concrete pore solution varies in the field. The effects of the varied alkalinity of a simulated solution on the durability of HFRP bars should be investigated in the future. It is worth noting that the bare HFRP bars cannot be directly applied in water. The deterioration of HFRP bars in water is more severe than that of GFRP bars.

## 4. Conclusions

This work investigates the effect of water immersion on the interlaminar shear strength of the GFRP bars and the HFRP bars and compares the impact of the carbon fiber coat on the durability of the mechanical properties of the GFRP bars immersed in water and alkaline solution. Moreover, it examines the mechanism for the improvement in the durability of the mechanical properties of the GFRP bars by the wrapped carbon fiber coat.

The diffusivity coefficient and saturation water absorption of the HFRP bars are larger than those of the GFRP bars. Thus, the carbon fiber coat of the HFRP bars enhances the diffusion of water into them. The thin carbon fiber coat neither changes the interlaminar shear strength of the GFRP bars nor has a positive effect on the improvement in the durability of the interlaminar shear strength of the GFRP bars immersed in water. For the specimens immersed in water for 140 days at a temperature of 21 and 40 °C, the interlaminar shear strength of the GFRP bars increases further than that of the HFRP bars. For the specimens immersed in water for 140 days at a temperature of 60 °C, the reduction in the interlaminar shear strength of the GFRP bars is less than that of the HFRP bars. The carbon fiber coat can efficiently enhance the durability of the interlaminar shear strength of the GFRP bars in the alkaline solution rather than in water. The retention of the interlaminar shear strength of the HFRP bars is higher than that of the GFRP bars at a similar immersion period.

## Figures and Tables

**Table 1:** The diffusion coefficient and saturation water absorption of the FRP bars fitted by the Equation (2).

Temperature (°C)	GFRP Bars		HFRP Bars	
	*D*_r_ (×10^–13^ m^2^/s)	*M*_∞_ (%)	*D*_r_ (×10^–13^ m^2^/s)	*M*_∞_ (%)
21	1.19	0.29	4.30	0.33
40	1.61	0.29	7.20	0.33
60	7.17	0.29	7.98	0.33

**Table 2:** The radial diffusivity coefficient of the carbon fiber coat calculated at various temperatures.

Temperature (°C)	*D*_rc_ ^a^ (×10^–13^ m^2^/s)	*M*_∞_ (%)
21	5.95	0.29
40	16.10	0.29
60	7.17	0.29

^a^ *D*_rc_ is the radial diffusivity coefficient of the carbon fiber coat.

## Data Availability

All data are available in the main text.
